# Evaluation of the irritable bowel syndrome severity index in Japanese male patients with irritable bowel syndrome with diarrhea

**DOI:** 10.1186/s13030-017-0092-x

**Published:** 2017-03-11

**Authors:** Motoko Ida, Akito Nishida, Hiraku Akiho, Yoshihiro Nakashima, Kei Matsueda, Shin Fukudo

**Affiliations:** 1 Japan-Asia Planning & Administration, Medical & Development, Astellas Pharma Inc., 2-5-1 Nihonbashi-Honcho, Chuo-ku, Tokyo, 103-8411 Japan; 2 Development Project Management, Astellas Pharma Inc., Tokyo, Japan; 3 Japan-Asia Clinical Development 2, Development, Astellas Pharma Inc., Tokyo, Japan; 4 Japan-Asia Data Science, Development, Astellas Pharma Inc., Tokyo, Japan; 5 Sakura Life Clinic, Tokyo, Japan; 6 Department of Behavioral Medicine, Tohoku University Graduate School of Medicine, Sendai, Japan

**Keywords:** 5-hydroxytryptamine (5-HT), Abdominal pain, Abdominal discomfort, Global improvement, Stool consistency, Irritable bowel syndrome severity index

## Abstract

**Background:**

Previous studies have indicated that ramosetron, a 5-hydroxytryptamine-3 receptor antagonist, achieves global improvement in irritable bowel syndrome (IBS) symptoms in male patients with IBS with diarrhea (IBS-D). However, in addition to global assessment it was deemed important to assess “clinically meaningful improvements, focusing on the patient’s chief complaint and the severity of major IBS symptoms”. We performed a randomized, placebo-controlled, phase IV pilot study to explore and examine efficacy variables that allow such evaluation of ramosetron in male patients with IBS-D.

**Methods:**

We performed a prospective study of 115 male outpatients with IBS-D (according to the Rome III criteria), from June 2009 to December 2009 at 25 centers in Japan. After a one-week baseline period, subjects received either 5 μg of ramosetron (*n* = 47) or placebo (*n* = 51) once daily for 12 weeks. To evaluate “clinically meaningful improvements focusing on the severity of major IBS symptoms,” the Japanese version of the IBS severity index (IBSSI-J) was used.

**Results:**

Change in IBSSI-J overall score from baseline was −133.5 ± 110.72 in the ramosetron 5 μg group and −108.2 ± 94.44 in the placebo group (*P* = 0.228) at the last evaluation point. Differences in responder rates for at least a 50% reduction from baseline in IBSSI-J between the ramosetron 5 μg group and the placebo group were over 10%, except Month 1. The monthly responder rate for global assessment of relief of overall IBS symptoms in the ramosetron 5 μg group showed a statistically significant improvement compared to placebo at the second month (44.4% vs 18.4%, *P* = 0.012). The proportion of patients who had a ≥ 50% reduction in IBSSI-J overall score was 24/37 (64.9%) in the responder group on global assessment and 18/54 (33.3%) in the non-responder group at Week 12.

**Conclusions:**

Further examination will be needed before IBSSI-J can be used in clinical trials of agents for IBS-D. However, this study revealed that response on global assessment was correlated with improvement in the IBSSI-J, suggesting that global assessment reflects improvement of the symptom severity of patients with IBS-D. (Clinicaltrials.gov ID: NCT00918411 Registered 9 June 2009).

## Background

Irritable bowel syndrome (IBS) is a functional gastrointestinal disorder that is characterized by chronic or recurrent abdominal pain and/or abdominal discomfort associated with abnormal bowel movements [1]. IBS as defined by the Rome III criteria [2] is classified into four subtypes: IBS with diarrhea (IBS-D), IBS with constipation (IBS-C), mixed-type IBS, and unsubtyped IBS. The onset of IBS and its symptoms have for some time been known to be largely associated with various psychosocial stressors. Psychosocial stress causes stimulation of the hypothalamus, releasing corticotropin-releasing hormone (CRH), and causes abnormalities in gastrointestinal motility and lowering of the sensory threshold in the gastrointestinal tract via neurotransmitters, such as 5-hydroxytryptamine (5-HT, serotonin) released from enteric nerves or enterochromaffin cells. Some evidence suggests that 5-HT has a crucial role in IBS-D pathophysiology. Patients with IBS-D show exaggerated colonic motility in response to colonic distention [3] and secretion of 5-HT [4]. Moreover, in animal studies and clinical pharmacological tests, 5-HT_3_ receptor antagonists have been reported to suppress abnormalities of gastrointestinal motility (abnormal bowel movements) and a decrease in the sensory threshold in the gastrointestinal tract caused by CRH and stresses [5–7], which suggests involvement of the 5-HT_3_ receptor in the occurrence of IBS-D symptoms.

Ramosetron, a potent, selective 5-HT_3_ receptor antagonist [5, 8–10], was developed in Japan, initially for the nausea and vomiting of cancer patients receiving chemotherapy and later for IBS-D patients. In clinical studies of IBS-D [11, 12], it was decided that abdominal pain and discomfort, which were the main subjective symptoms of patients with IBS-D, would be assessed by subjects as “global assessment of relief of abdominal pain/discomfort” and that symptoms of diarrhea, such as abnormal stool form, frequent bowel movement and defecation urgency, would be evaluated by patients as “global assessment of improvement in abnormal bowel habits”. Furthermore, all subjective symptoms that patients had, including the above symptoms, were comprehensively assessed by the patients as “global assessment of relief of overall IBS symptoms”. Of these three global assessments of improvement/relief, the “global assessment of relief of overall IBS symptoms” in particular was considered to be directly linked to therapeutic effects in patients with IBS-D because of the heterogeneity of symptoms, so it was adopted as the primary variable for clinical studies of ramosetron. The efficacy of ramosetron was demonstrated based on the results of this variable in a previous phase III study. However, stratified analysis by sex using the chi-square test (two-sided significance level of 0.05) in the phase III study revealed that ramosetron did not show significant improvement compared to placebo in the global assessment of relief of the overall IBS symptoms of female patients [12]. Based on the above results, marketing approval was granted for the indication of “IBS-D in male patients” in Japan in July 2008. Subsequently, additional clinical studies [13–15] were conducted to evaluate the efficacy and safety of ramosetron for female patients with IBS-D. These studies indicated that 2.5 μg/day of ramosetron was an effective treatment for female patients with IBS-D, in contrast to the optimal dose of ramosetron at 5 μg/day for male patients. Ramosetron was approved for use by women in May, 2015.

The Pharmaceuticals and Medical Devices Agency of Japan has approved the use of global assessment as a primary endpoint for IBS studies since 2002 [11, 12]. However, they also considered it important to assess “clinically meaningful improvements, focusing on the patient’s chief complaint and the severity of major IBS symptoms” in addition to the global assessment. The aim of this study was to explore and examine variables that allow such evaluation of ramosetron in patients with IBS-D. The IBS severity index (IBSSI) is a reliable and well-validated instrument for measuring the presence and severity of specific IBS symptoms [16]. Japanese versions of the IBS severity index (IBSSI-J) developed and validated by Shinozaki et al. are available in Japan [17]. Most studies confirming responsiveness of IBSSI were trials aiming at evaluating behavioral interventions. Preliminary evaluation was thought to be needed to assess responsiveness of IBSSI-J in clinical trials using pharmacological agents. This study was conducted as a pilot study for a post marketing study.

## Methods

### Patient population

This study was conducted from June 2009 to December 2009 at 25 Japanese centers that have departments of gastroenterology. Male outpatients aged 20–64 years were diagnosed with IBS-D based on the Rome III criteria. The study protocol was designed in accordance with the Declaration of Helsinki and was approved by the institutional review board at each site. All patients provided written informed consent prior to participating in study-related procedures.

In the Rome III criteria [2], IBS-D is defined as recurrent abdominal pain/discomfort for at least three days per month in the preceding three months, in association with two or more of the following: improvement with defecation, onset associated with a change in the frequency of stools, and/or onset associated with a change in the form (appearance) of stools. Furthermore, patients have loose (mushy) or watery stools at least 25% of the time and hard or lumpy stools for less than 25% of bowel movements.

Patients were eligible if they fulfilled the criteria for the last three months, with symptom onset at least six months prior to diagnosis. Organic diseases were excluded by colonoscopy or double-contrast barium enema if these examinations had not been performed within five years. Based on a medical interview conducted by the attending physician before provisional registration, patients were excluded if any of the following were evident: a history of resection of the stomach, small intestine, or large intestine (excluding appendicitis or resection of benign polyps); history or current evidence of inflammatory bowel disease; history or current evidence of ischemic colitis, concurrent infectious enteritis, hyperthyroidism, hypothyroidism, or other diseases that may affect gastrointestinal transit or colonic function; history or current evidence of abuse of drugs or alcohol within the previous year; malignant tumors; current evidence of severe depression or a severe anxiety disorder that could potentially affect the evaluation of study drug efficacy; concurrent serious cardiovascular, respiratory, renal, hepatic, gastrointestinal (excluding IBS), hematological, or neurological/psychiatric diseases; or a history of drug allergies. In addition, patients were excluded if they were using drugs or undergoing examinations that could affect the evaluation of study drug efficacy; if they had been enrolled in previous clinical studies of ramosetron or had taken ramosetron; and if they were participating or had participated in other clinical studies within the 12 weeks prior to study initiation.

Patients satisfying the inclusion and exclusion criteria for typical IBS-D symptoms during a one-week baseline period were enrolled. Severity of abdominal pain/discomfort had to exceed mean scores of 0.7 or more assessed daily on a 5-point ordinate (numerical rating) scale (0, none; 1, mild; 2, moderate; 3, severe; and 4, intolerable). The number of bowel movements had to exceed three times or more per week. Stool consistency was assessed with using the Bristol Stool Form Scale (BSFS) [2] as follows; type 1, separate hard lumps, like nuts (hard to pass); type 2, sausage shaped but lumpy; type 3, like a sausage but with cracks on its surface; type 4, like a sausage or snake, smooth and soft; type 5, soft blobs with clear-cut edges (passed easily); type 6, fluffy pieces with ragged edges (mushy stool); or type 7, watery, no solid pieces, and entirely liquid. Following this classification of stool consistency using the BSFS, patients who had either type 1 or type 2 stools were excluded. Patients who had not used drugs or undergone examinations that could affect the evaluation of study drug efficacy within 10 days prior to randomization; who recorded all items in the patient diary for five days or more during the baseline period; and who were not judged ineligible for the study according to the clinical laboratory test results obtained before the baseline period were randomized and then given treatment.

### Study design

This randomized, placebo-controlled clinical trial comprised a provisional registration period, a one-week baseline period, and a 12-week treatment period, similar to previous studies [11, 12]. Following the baseline period, eligible patients were randomly assigned to 12-week oral treatment with placebo or ramosetron hydrochloride 5 μg once daily before breakfast. Visits were scheduled at Weeks 2, 4, 8, and 12 (or at discontinuation) to assess treatment efficacy, drug compliance, and occurrence of adverse events. Randomization was performed in a 1:1 ratio using a block size of four based on a randomization list developed by a third-party contract research organization. Placebo tablets were externally distinguishable from ramosetron hydrochloride tablets, however, they were indistinguishable when packaged in press through pack sheets. Patients were prohibited to use drugs or undergo examinations, such as other IBS therapeutic drugs, antidiarrheal drugs, and colonoscopy, that could affect the evaluation of study drug efficacy during the treatment period. All patients, investigators, and sponsors were blinded until all observations and evaluations were completed, the statistical analysis plan was finalized, and all data had been locked. All authors had access to the study data and reviewed and approved the final manuscript.

### Data collection

During the baseline and treatment periods, patients recorded their IBS symptoms daily on paper diary cards at bedtime. In the diary, patients recorded the BSFS for every bowel movement throughout the study period. Patients scored severity on a five-point ordinate (numerical rating) scale and the duration of all continuous abdominal pain/discomfort from Week 1 to Week 4, Week8 and Week12 they had experienced. Every seven days during the treatment period, patients also graded summarized IBS symptoms compared with the baseline period on a five-point ordinate scale as follows: relief from overall IBS symptoms and abdominal pain/discomfort (0, completely relieved; 1, considerably relieved; 2, somewhat relieved; 3, unchanged; and 4, worsened) and improvement in abnormal bowel habits (0, nearly normalized; 1, considerably relieved; 2, somewhat relieved; 3, unchanged; and 4, worsened). Patients assessed IBS severity using the Japanese version of the IBS Severity Index (IBSSI-J) every four weeks [16, 17].

### Efficacy and safety endpoints

To explore and examine variables that allow evaluation of “clinically meaningful improvements, focusing on the severity of major IBS symptoms” achieved by this drug, the IBSSI-J was assessed as a new measure in this trial in addition to the previous global assessment [11, 12]. IBSSI-J contains five questions that measure, on a 100-point scale, the severity of abdominal pain, the frequency of abdominal pain, the intensity of abdominal distention, dissatisfaction with bowel habits, and interference with QOL. All five components contribute to the score equally, yielding overall scores ranging from 0 to 500. IBS severity is graded as mild (75–174), moderate (175–299), or severe (300–500) on the basis of overall scores [16]. Patients who had at least a 50% reduction from baseline (≥50% reduction) in IBSSI-J overall score were defined as responders at each evaluation point. In the monthly responder rates for global assessment of relief of overall IBS symptoms, relief of abdominal pain/discomfort and improvement in abnormal bowel habits, patients with scores of 0 or 1 at each weekly evaluation point were defined as weekly responders, and patients who were weekly responders for at least two of the four weeks were defined as monthly responders. Change in IBSSI-J score and percent change in the IBSSI-J from baseline were calculated with reference to the category of global assessment of overall IBS symptoms (responder vs. non-responder) at each evaluation point.

### Statistical analysis

Sample sizes of 60 patients or more (30 patients/group or more) were set based on the feasibility of a post marketing study to explore and examine the endpoints of the patient’s chief complaint or IBS severity. Statistical analysis was performed using SAS Drug Development (ver. 3.4) and PC-SAS (ver. 8.2) (SAS Institute Inc., Cary, NC, USA).

Efficacy analyses included the full analysis set (FAS), which was as complete as possible and as close as possible to the intention-to-treat ideal of including all randomized subjects. The FAS included all patients who received at least one dose of the study drug during the treatment period and for whom at least one endpoint could be evaluated. To determine the robustness of the results, primary analyses were performed according to the per-protocol set. Safety analyses were performed for all patients who received at least one dose of the study drug during the treatment period.

Change from baseline and percent change from baseline in IBSSI-J score were summarized at each evaluation point by treatment group and/or monthly response for global assessment of relief of overall IBS symptoms. Treatment comparison used a *t*-test with a two-sided significance level of 0.05. IBSSI score was categorized and summarized by whether the subject was a monthly responder on global assessment of relief of overall IBS symptoms.

Monthly responder rates for global assessment of relief of overall IBS symptoms are expressed as a percentage of responders, and 95% confidence intervals (95% CIs) are presented. The treatment groups were compared using the chi-square test with a two-sided significance level of 0.05. In addition, responder rates for at least a 50% reduction from baseline (≥50% reduction) in IBSSI-J at each evaluation point are similarly analyzed as an ad hoc analysis.

## Results

### Overall study population

**Fig. 1:**
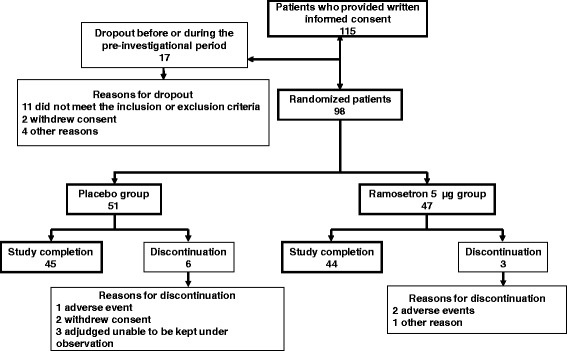
Flowchart showing patient progress throughout the study. Reasons for dropping out of the study are shown

Written informed consent was provided by 115 patients. Of these, 17 patients dropped out and 98 patients were randomly allocated to the ramosetron 5 μg group (*n* = 47), or the placebo (*n* = 51) group (Fig. 1). Ultimately 44 patients in the ramosetron 5 μg group and 45 patients in the placebo group completed the study. The reasons for discontinuation are shown in Fig. 1. In the placebo group, one patient discontinued by withdrawing consent after randomization, with no data, and was excluded from the FAS used in the efficacy analyses. The decision to exclude this patient from FAS was taken before unblinding, according to the predefined procedure stipulated in the study protocol.

All the demographic and baseline characteristics shown in Table 1 were similar among patients allocated to each group. The medication adherence rates were 97.6% in the ramosetron 5 μg group and 97.9% in the placebo group.

**Table 1:** Demographics and baseline characteristics

Patient background	Placebo	Ramosetron 5 μg	*P* value
	(*n* = 50)	(*n* = 47)	
Age (years)	40.9 ± 11.11	41.0 ± 9.31	0.970
Duration of disease (months)	103.9 ± 90.27	111.5 ± 129.10	0.738
Severity of abdominal pain/discomfort (0–4)	1.43 ± 0.58	1.52 ± 0.61	0.481
Bristol Stool Form Scale (1–7)	5.55 ± 0.66	5.52 ± 0.43	0.764
Stool frequency (times/day)	2.77 ± 1.33	2.44 ± 1.09	0.181
IBSSI-J overall score	246.6 ± 80.52	267.1 ± 98.75	0.264
No symptoms (0–74)	0 (0.0%)	1 (2.1%)	-
Mild (75–174)	7 (14.0%)	10 (21.3%)	-
Moderate (175–299)	32 (64.0%)	14 (29.8%)	-
Severe (300–500)	11 (22.0%)	22 (46.8%)	-

Data are expressed as mean ± standard deviation. *P* values were calculated using analysis of variance

### Efficacy

The baseline IBSSI-J overall scores in the ramosetron 5 μg and placebo groups were 267.1 ± 98.75 and 246.6 ± 80.52, respectively (Table 2). The respective first-quartile point and third-quartile points were 180.0 and 355.0 in the ramosetron 5 μg group and 200.0 and 290.0 in the placebo group. Severity of IBS can be graded as mild (75–174), moderate (175–299), or severe (300–500) on the basis of overall IBSSI scores. The proportions of patients with moderate severity at baseline were 29.8% in the ramosetron 5 μg group and 64.0% in the placebo group, with severe grading 46.8 and 22.0%, respectively (Table 1). Most patients enrolled in this study were classified as moderate to severe. Table 2 also shows the baseline score for each of the five components included in the IBSSI-J. The highest score was dissatisfaction with bowel habits, 68.6 ± 25.55 and 66.8 ± 22.78 in the ramosetron 5 μg and placebo groups, respectively. Second was interference with QOL (60.0 ± 27.59 and 54.3 ± 27.39, respectively), followed by frequency of abdominal pain (55.1 ± 33.87 and 57.4 ± 33.61, respectively). Intensity of abdominal distention showed the lowest scores, 35.6 ± 32.25 and 23.8 ± 25.65, respectively. Abdominal pain was assessed from the aspects of severity and frequency in the IBSSI-J. Frequency of abdominal pain was worse than severity of abdominal pain.

**Table 2:** Baseline IBSSI-J score

		N	Mean ± SD	Min	Max	Median	First-quartile points	Third-quartile points	*t*-test
Overall score	Placebo	50	246.6 ± 80.52	80	410	245	200	290	t = −1.123, df = 95, *P* = 0.264
	Ramosetron 5 μg	47	267.1 ± 98.75	60	440	275	180	355	
Severity of abdominal pain	Placebo	50	44.3 ± 26.71	0	100	47.5	20	70	t = −0.647, df = 95, *P* = 0.519
	Ramosetron 5 μg	47	47.8 ± 26.70	0	90	50	30	70	
Frequency of abdominal pain	Placebo	50	57.4 ± 33.61	0	100	60	30	90	t = 0.335, df = 95, *P* = 0.739
	Ramosetron 5 μg	47	55.1 ± 33.87	0	100	60	30	90	
Intensity of abdominal distention	Placebo	50	23.8 ± 25.65	0	80	17.5	0	50	t = −2.007, df = 95, *P* = 0.048
	Ramosetron 5 μg	47	35.6 ± 32.25	0	100	30	0	60	
Dissatisfaction with bowel habits	Placebo	50	66.8 ± 22.78	20	100	60	50	90	t = −0.361, df = 95, *P* = 0.719
	Ramosetron 5 μg	47	68.6 ± 25.55	0	100	70	50	90	
Interference with QOL	Placebo	50	54.3 ± 27.39	0	100	55	30	80	t = −1.013, df = 95, *P* = 0.314
	Ramosetron 5 μg	47	60.0 ± 27.59	0	100	60	40	80	

Data are expressed as mean ± standard deviation. *P* values were calculated using analysis of variance

**Fig. 2:**
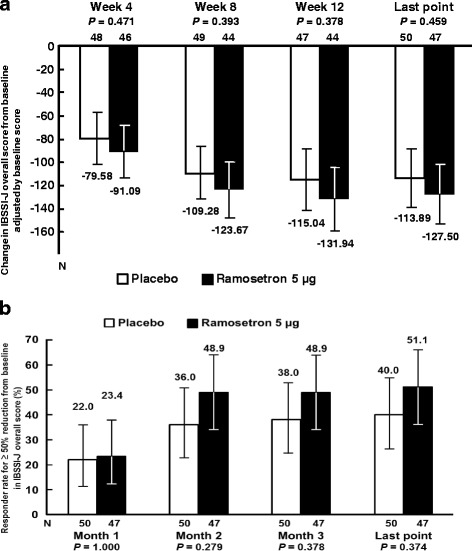
Change in IBSSI-J overall scores. **a** Change in IBSSI-J overall scores from baseline, adjusted by baseline score. Column height: the values adjusted using the baseline score as a covariate. Error bar: 95% CI. *P* values were calculated using analysis of covariance with the treatment group as a factor and baseline score as a covariate. **b** Responder rates for at least a 50% reduction from baseline in IBSSI-J overall score. Column height: responder rate (%). Error bar: 95% CI

Change in IBSSI-J overall score from baseline (Table 3) was −133.5 ± 110.72 in the ramosetron 5 μg group and −108.2 ± 94.44 in the placebo group (*P* = 0.228) at the last evaluation point. Differences between the ramosetron 5 μg and placebo groups adjusted by baseline scores were −11.51 (95% CI, −43.13–20.11, *P* = 0.471) at Week 4, −14.39 (95% CI, −47.70–18.93, *P* = 0.393) at Week 8, −16.90 (95% CI, −54.80–21.01, *P* = 0. 378) at Week 12 and −13.60 (95% CI, −49.89–22.68, *P* = 0.459) at the last evaluation point (Fig. 2a). Differences in responder rates for at least a 50% reduction from baseline (≥50% reduction) in IBSSI-J between in the ramosetron 5 μg group and the placebo group were over 10%, except Month 1 (Fig. 2b). Changes in each component of the IBSSI-J from baseline in the ramosetron 5 μg and placebo groups at all evaluation points are shown in Table 3.

**Table 3:** Change in each IBSSI-J component score from baseline at each evaluation point

	Week 4		Week 8		Week 12		Last point	
	Placebo	Ramosetron	Placebo	Ramosetron	Placebo	Ramosetron	Placebo	Ramosetron
		5 μg		5 μg		5 μg		5 μg
	Mean ± SD	Mean ± SD	Mean ± SD	Mean ± SD	Mean ± SD	Mean ± SD	Mean ± SD	Mean ± SD
	*P* value	*P* value	*P* value	*P* value	*P* value	*P* value	*P* value	*P* value
N	48	46	49	44	47	44	50	47
Overall scores	−75.0 ± 81.52	−95.9 ± 105.12	−103.0 ± 81.81	−130.6 ± 114.27	−110.3 ± 97.04	−137.0 ± 113.18	−108.2 ± 94.44	−133.5 ± 110.72
		*P* = 0.283		*P* = 0.181		*P* = 0.231		*P* = 0.228
Severity of abdominal pain	−15.3 ± 25.18	−17.0 ± 25.15	−21.4 ± 28.46	−24.3 ± 27.41	−21.9 ± 30.42	−26.6 ± 27.01	−21.9 ± 29.76	−26.1 ± 26.41
		*P* = 0.743		*P* = 0.625		*P* = 0.439		*P* = 0.467
Frequency of abdominal pain	−16.5 ± 25.89	−20.0 ± 32.52	−24.7 ± 29.38	−26.6 ± 35.04	−25.1 ± 32.56	−28.2 ± 35.13	−24.6 ± 31.77	−28.1 ± 33.98
		*P* = 0.560		*P* = 0.777		*P* = 0.666		*P* = 0.603
Intensity of abdominal distension	−9.7 ± 23.13	−16.3 ± 27.68	−12.2 ± 24.54	−17.8 ± 26.66	−11.5 ± 25.83	−16.6 ± 29.88	−10.8 ± 25.26	−16.3 ± 29.22
		*P* = 0.214		*P* = 0.295		*P* = 0.385		*P* = 0.325
Dissatisfaction with bowel habits	−16.2 ± 31.23	−20.8 ± 28.18	−19.2 ± 33.55	−31.4 ± 29.88	−25.1 ± 33.57	−33.4 ± 31.50	−24.8 ± 33.84	−32.5 ± 30.63
		*P* = 0.454		*P* = 0.067		*P* = 0.230		*P* = 0.243
Interference with QOL	−17.3 ± 27.76	−21.8 ± 29.94	−25.5 ± 27.75	−30.5 ± 34.84	−26.7 ± 28.67	−32.2 ± 32.53	−26.1 ± 28.04	−30.6 ± 32.44
		*P* = 0.454		*P* = 0.445		*P* = 0.396		*P* = 0.470

Data are expressed as mean ± standard deviation. *P* values were calculated using analysis of variance

**Fig. 3:**
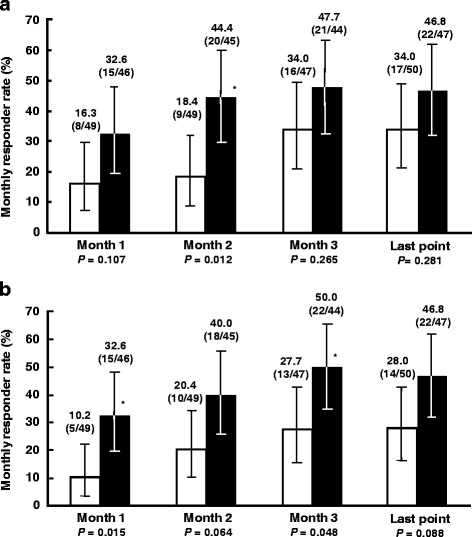
Global assessments. **a** Monthly responder rates for global assessments of relief of overall IBS symptoms. **b** Monthly responder rates for improvement in abnormal bowel habits. Column height: responder rate (%). Error bar: 95% CI. *P* values were calculated using the chi-square test, as follows: *P < 0.05

The monthly responder rate for global assessment of relief of overall IBS symptoms at the last evaluation point was 46.8% (95% CI, 32.1–61.9) in the ramosetron 5 μg group and 34.0% (95% CI, 21.2–48.8, *P* = 0.281) in the placebo group (Fig. 3a). Even though the number of patients enrolled in this study is limited, a statistically significant difference between ramosetron and placebo was shown at the second month (*P* = 0.012). Monthly responder rates for improvement in abnormal bowel habits in the ramosetron 5 μg group were significantly higher than those in the placebo group at the first month (*P* = 0.015) and the third month (*P* = 0.048) (Fig. 3b). On the other hand, monthly responder rates for abdominal pain/discomfort in the ramosetron 5 μg group did not show a statistically significant difference between ramosetron and placebo at any evaluation point (data not shown).

**Fig. 4:**
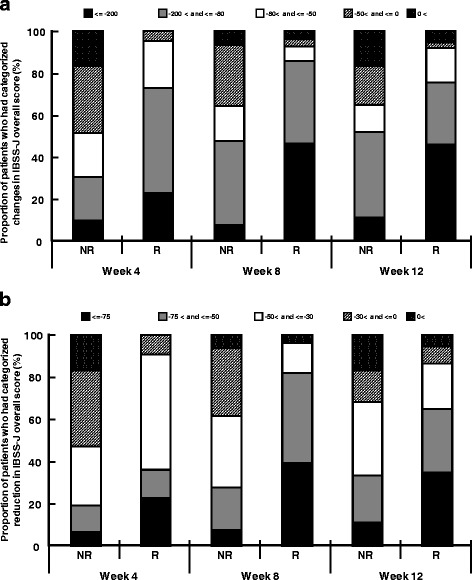
Relationship between IBSSI-J and global assessment. **a** Changes in IBSSI-J overall scores from baseline were compared by responder/non-responder for global assessment of relief of overall IBS symptoms. Mean changes in IBSSI-J overall scores from baseline were categorized into the following groups: ≤ −200, −200 < and ≤ −80, −80 < and ≤ −50, −50 < and ≤ 0, 0 <. **b** Percent change in IBSSI-J overall score from baseline was compared by responder/non-responder for global assessment of relief of overall IBS symptoms. Percent change in IBSSI-J from baseline was categorized into the following groups: ≤ −75, −75 < and ≤ −50, −50 < and ≤ −30, −30 < and ≤ 0 and 0 <

Figure 4 shows the relationship between IBSSI-J and the global assessment of relief of overall IBS symptoms. Mean changes in IBSSI-J overall scores from baseline are categorized into ≤ −200, −200 < and ≤ −80, −80 < and ≤ −50, −50 < and ≤ 0, 0 < and compared by responder/non-responder for global assessment of relief of overall IBS symptoms in Fig. 4a. Patients who had mean changes in IBSSI-J overall scores from baseline exceeding 200 points were more numerous in the responder group on global assessment compared to the non-responder group (45.9% vs 11.1% at Week 12). Patients with a change of over 80 points or over 50 points were also more numerous in the responder group on global assessment than in the non-responder group at all evaluation points. Similarly, the percent change in IBSSI-J from baseline was categorized into ≤ −75, −75 < and ≤ −50, −50 < and ≤ −30, −30 < and ≤ 0 and 0 < and compared by responder/non-responder for global assessment of relief of overall IBS symptoms (Fig. 4b). The number of patients who had a ≥ 75% reduction in IBSSI-J overall score was higher in the responder group on global assessment than in the non-responder group (35.1% vs 11.1% at Week 12). The rate of patients who had a ≥ 50% reduction or ≥ 30% reduction in IBSSI-J overall score was also higher in the responder group on global assessment than in the non-responder group at all evaluation points.

### Safety

Safety was evaluated for all 98 patients. Adverse events were experienced by 27 patients (57.4%) in the ramosetron 5 μg group and by 20 patients (39.2%) in the placebo group (Table 4). The incidence of hard stool was higher in the ramosetron 5 μg group than in the placebo group, which was considered to be caused by the pharmacological action of ramosetron. All the events including constipation and hard stool observed in this study were mild and improved quickly. There was no occurrence of ischemic colitis or serious adverse events.

**Table 4:** Incidence of adverse events

Event	Placebo	Ramosetron 5 μg
	(*n* = 51)	(*n* = 47)
All adverse events	20 (39.2%)	27 (57.4%)
Gastrointestinal disorders	8 (15.7%)	13 (27.7%)
Abdominal discomfort	0 (0.0%)	2 (4.3%)
Constipation	2 (3.9%)	0 (0.0%)
Hard stool	3 (5.9%)	9 (19.1%)
Nausea	2 (3.9%)	0 (0.0%)
Infections and infestations	4 (7.8%)	5 (10.6%)
Nasopharyngitis	4 (7.8%)	3 (6.4%)
Gastroenteritis	0 (0.0%)	2 (4.3%)
Hepatobiliary disorders	2 (3.9%)	2 (4.3%)
Hepatic function abnormal	2 (3.9%)	1 (2.1%)
Skin and subcutaneous tissue disorder	2 (3.9%)	3 (6.4%)
Dermatitis contact	1 (2.0%)	2 (4.3%)

Data are expressed as number (%). Events with an incidence of ≥ 3% in any of the groups are listed

## Discussion

In this clinical study, IBSSI-J was used to explore and examine variables that allow the assessment of “clinically meaningful improvements, focusing on the severity of major IBS symptoms”. We showed that most patients enrolled had moderate to severe IBS symptoms in the baseline period. The highest score among each component was for dissatisfaction with bowel habits. Second were interference with QOL and frequency of abdominal pain. It is well known that IBS significantly impairs health related quality of life (QOL) [18]. The patients in this study were considered to have impaired QOL. The lowest score for the five components was for intensity of abdominal distention. Patients with abdominal distention and/or bloating were reported to be more numerous with IBS-C than with IBS-D [19]. The lowest score of intensity of abdominal distention in IBSSI-J in this study might be related to a lower contribution of abdominal distention to IBS symptom severity in IBS-D.

The proportion of patients who had a ≥ 50% reduction in IBSSI-J overall score was more than 10% higher in the ramosetron 5 μg group than in the placebo group, except Month 1. Although significant results were lacking, the graph shape of changes in IBSSI-J score at all evaluation points in the ramosetron 5 μg group seems to be superior to that of the placebo group. Francis et al. suggested that a decrease of 50 points in IBSSI overall score correlated with improvement in clinical symptoms [16]. On the other hand, Whitehead et al. have proposed that ≥ 50% reduction in IBSSI overall score from the baseline score was considered to constitute clinically meaningful improvement of symptoms [20]. We evaluated other categorization to find ‘clinically meaningful improvements’ by pharmacological agents, and compared responder/non-responder for global assessment of relief of overall IBS symptoms. In Francis’s report, mean change of IBSSI from baseline to 3 months later was significantly greater in the patients who became clinically considerably better than little changed (change in score: 83 vs 6) [16]. Based on their reports, we selected −50 and −80 point reductions. The baseline IBSSI-J overall scores in our ramosetron 5 μg and placebo groups were 267.1 ± 98.75 and 246.6 ± 80.52, respectively (Table 1). Because IBS severity is rated as No symptoms (0–74), we set a −200 point reduction as the score at which the symptoms were eliminated. Similarly, in addition to 50% reduction, we examined 3/4 and 1/3 reduction categories to explore the clinical meaningful change. Our study showed patients who had changes in their overall IBSSI-J scores from baseline of over 50 points were more numerous in the monthly responder group based on global assessment of relief of overall IBS symptoms than in the non-responder group. This finding is in accordance with the results of Francis. The proportion of patients who had a ≥ 50% reduction in IBSSI-J overall score was also higher in the responder group on global assessment (24/37, 64.9%) than in the non-responder group (18/54, 33.3%) at Week 12. The studies by Francis et al. and Whitehead et al. were trials aiming to evaluate behavioral interventions, and these effects were not compared to placebo. In patients with IBS-C, it was recently reported that linaclotide showed a statistically significantly higher change in IBSSI overall score from baseline as well as in the percentage of patients with ≥ 50% reduction in IBSSI overall score compared to placebo [21]. Nevertheless, those data suggest that the IBSSI could be used for measuring response to pharmacological agents for patients with IBS-C; there is little data used for measuring the response of patients with IBS-D. This study was the first trial to use the IBSSI-J to measure the response to pharmacological agents in patients with IBS-D.

Despite the limited patient number in this study, statistically significant differences between ramosetron and placebo were shown in the monthly responder rate for global assessment of relief of overall IBS symptoms at the second month and in the monthly responder rates for improvement in abnormal bowel habits at the first and the third months. Improvement in bowel habits was shown to contribute to improvement of global assessment of relief of overall IBS symptoms, as in previous studies [12]. The differences between ramosetron and placebo were more evident than those in the IBSSI-J overall scores.

Whitehead et al. reported that patients with milder symptoms at baseline were more likely to report satisfactory relief than patients with moderate or severe symptoms in a usual care study [20]. On the other hand, Drossman et al. reported that baseline symptom severity was no longer confounded with a report of adequate relief (AR) at the study end point, if patients who reported AR at baseline were excluded from study participation [22]. A meta-analysis involving 10,066 IBS patients to investigate whether improvement of symptoms depends on their severity showed no correlation between the severity of IBS and the improvement of symptoms in a binary assessment [23]. In this study, the sample size of each severity group was too small to permit assessment of the relationship between baseline severity and global assessment. AR was not used.

In this study, the monthly responder group with respect of global assessment of relief of overall IBS symptoms showed a greater change in the IBSSI-J overall score and percent change from baseline than did the non-responder group. This study thus revealed that responses on global assessment were correlated with improvement in IBSSI-J, suggesting that global assessment reflects improvement of the symptom severity of patients with IBS-D. IBS is a syndrome that includes multiple symptoms (abdominal pain/discomfort, stool form, stool frequency, etc.) [1]. Global assessments of relief of overall IBS symptoms allow patients to assess improvement in multiple IBS symptoms [11–15]. The Japanese Society of Gastroenterology (JSGE) developed evidence-based clinical practice guidelines for IBS [24]. They recommend treating IBS patients as they can feel improvement in IBS symptoms based on the assessment of patient-reported outcomes. Global assessment of relief of overall IBS symptoms can be a useful efficacy variable in IBS-D.

IBSSI-J showed that the patients in this study have impaired QOL. For the improvement of QOL, it is important for IBS patients to evaluate ‘clinically meaningful improvements’, because it was reported IBS patients impaired their QOL [18]. Although generic QOL instruments like SF-36 can be good measurement tools for comparing the impact of different conditions on health status, disease-specific QOL is considered to be sensitive for measuring the impact of treatment. IBS-QOL is a reliable and well-validated outcome for assessing the QOL of IBS patients. We used IBS-QOL and obtained the result that ramosetron significantly improved IBS-QOL compared to placebo in the following post marketing study for male patients with IBS-D [25].

This study was a pilot study and has some limitations. First, the sample size was not sufficient to detect a statistically significant difference between ramosetron and placebo. Second, this study was conducted with only male patients with IBS-D. Third, psychosocial factors may have affected to the response to the drug. Especially, expectations of the drug efficacy, which differs among patients, is likely to affect the drug effects [26]. The effect of expectations can be tested by use of placebo [26]. Therefore, the drug effect seen in clinical trials are supposed to be a summation of placebo effects and real pharmacological effects. Because we included a placebo group in this study, it is easier to assume a real pharmacological effect of ramosetron. Further experience will be needed to use this questionnaire as a primary endpoint in clinical studies related to the development of the pharmacological agents for IBS-D patients.

## Conclusions

Further examination will be needed before IBSSI-J can be used in clinical trials of agents for IBS-D. However, this study revealed that the responses on global assessment were correlated with improvement in the IBSSI-J, suggesting that global assessment reflects improvement of the symptom severity of patients with IBS-D.

## Abbreviations

BSFS: Bristol Stool Form Scale
95% CI: 95% confidence interval
CRH: Corticotropin-releasing hormone
FAS: Full analysis set, 5-HT, 5-hydroxytryptamine
5-HT_3_: 5-hydroxytryptamine receptor 3
IBS: Irritable bowel syndrome
IBS-C: Irritable bowel syndrome with constipation
IBS-D: Irritable bowel syndrome with diarrhea and IBSSI-J, Japanese version of IBS Severity Index
